# Successful diagnosis of humidifier lung by individual provocation test to a responsible environment, a case report

**DOI:** 10.1016/j.rmcr.2022.101639

**Published:** 2022-03-22

**Authors:** Takumi Murakami, Yuki Iijima, Takahiro Ando, Masaru Ejima, Tsuyoshi Shirai, Haruhiko Furusawa, Tsukasa Okamoto, Tomoya Tateishi, Meiyo Tamaoka, Yasunari Miyazaki

**Affiliations:** Department of Respiratory Medicine, Tokyo Medical and Dental University, Tokyo, Japan

**Keywords:** Humidifier lung, Provocation test, Hypersensitivity pneumonitis

## Abstract

A 52-year-old woman presented with repeating episodes of pneumonia which spontaneously resolved after hospitalization. Hypersensitivity pneumonitis was suspected, but the causative antigen was not determined whether the parakeets she kept or the humidifier she owned was causative exposure. To identify which exposure is culprit, individual provocation test to a responsible environment was sequentially conducted. First, a home-returning provocation test to the parakeet was negative. Contrarily, the humidifier provocation test to her own humidifier was positive for symptoms, radiological changes, and inflammatory responses in blood test. Finally, she was diagnosed as having humidifier lung. When several antigens are suspected to be the causative agents for hypersensitivity pneumonitis, a step-by-step provocation tests is useful.

## 1 Introduction

Antigen avoidance is mainstay for managing hypersensitivity pneumonitis (HP) because an insufficient avoidance may result in continuous exposure and development of fibrosis [1,2]. Although the identification of a causative antigen is needed for avoidance, it is sometimes challenging because diversity of inciting agents is known to cause HP [3]. While a thorough medical history can elucidate several antigens, it is difficult to specify which one is a culprit exposure for the disease. In such a situation, provocation tests are helpful to solve these problems. Here, we present a case of humidifier lung in which a combination of provocation tests was successful for diagnosis.

## 2 Case report

**Fig. 1:**
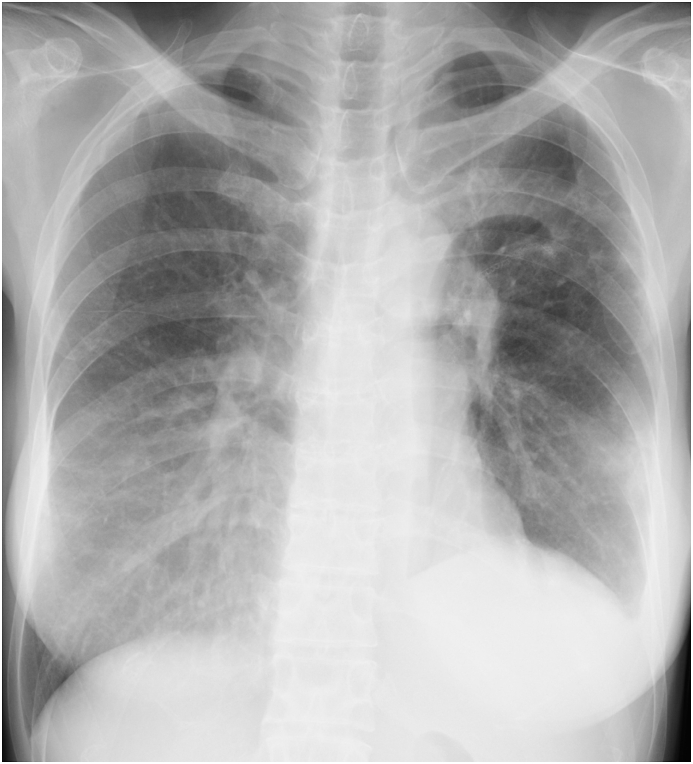
Chest radiography during the previous hospital admission.

**Fig. 2:**
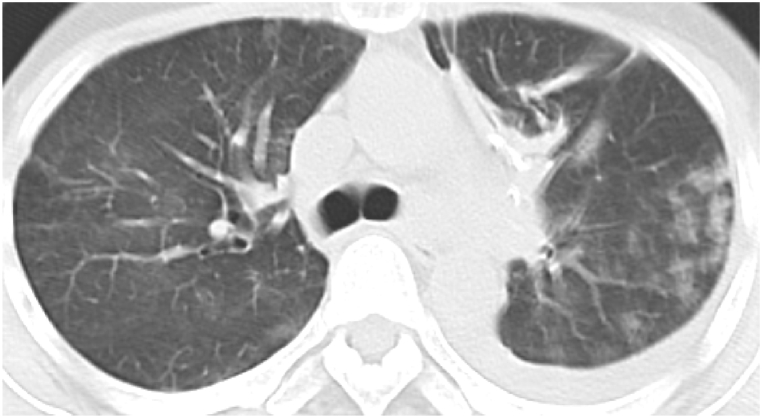
Chest CT during the previous hospital admission.

**Fig. 3:**
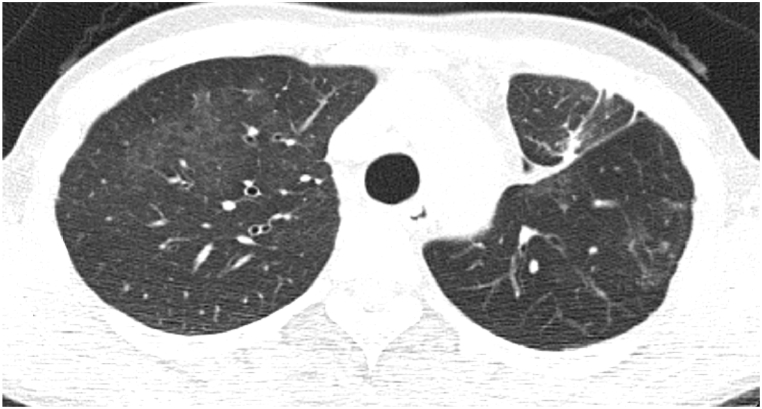
Chest CT during the second hospitalization for pneumonia.

A 52-year-old woman was referred to our hospital for examination of a two-month history of recurrent pneumonia. Two months before presentation to our hospital, she had been admitted to another hospital for resection of a benign lung nodule in the left upper lung. After discharge from the hospital, she was soon readmitted because of acute respiratory failure with worsening cough and fever. Chest X-rays showed ground glass nodules in the left upper lung field (Fig. 1). A chest computed tomography (CT) showed a centrilobular ground glass nodules and mosaic attenuation bilaterally (Fig. 2), but they spontaneously resolved after readmission. After discharge from the hospital, pneumonia had flared up again (Fig. 3), which also recovered only by hospitalization. HP was suspected, and she was introduced to our hospital. She had a medical history of hypothyroidism, but medication was not needed. She had 28 pack-year of smoking history. Since childhood, she had taken care of parakeets and kept close contact during sleep and feeding by mouth. She had used a down jacket for several years. She had put a new ultrasonic humidifier 3 months before hospitalization, but the cleaning of the humidifier tank was not adequate.

On admission, she had no fever, and transcutaneous arterial oxygen saturation (SpO_2_) was 95% (room air). Fine crackles were auscultated at the left anterior chest. White blood cell (WBC) count was 8100/μL, C-reactive protein (CRP) was 0.04 mg/dL, and Krebs von Lungen 6 (KL-6) was 273 U/ml, which were all within normal ranges, while Surfactant Protein D (SP-D) was elevated to 159 ng/ml. Anti *Trichosporon Asahii* antibody and anti-pigeon dropping extracts (anti-PDE) antibodies were negative. However, the lymphocyte stimulation test (LST) against pigeon serum was positive at 262% of the stimulation index.

**Fig. 4:**
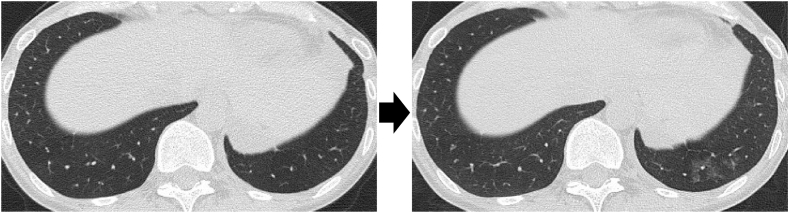
Changes in the Chest CT after the humidifier provocation test.

The history of having close contact with the parakeets and the use of the humidifier were suspected to be potential causal exposures of HP. To identify the culprit antigen, a combination of provocation tests was planned. Namely, two different challenge tests, the home-returning provocation test and the humidifier provocation test were sequentially conducted. During the half-day home-returning provocation test, the patient was instructed to have contact with the parakeets as usual, but to avoid using the humidifier. However, no obvious change was observed in any of the symptoms, serum inflammatory response, body temperature, or radiographic changes (Table 1). Subsequently, the humidifier provocation test was conducted. In a hospital room, the patient was exposed to her own humidifier for one hour. Consequently, fever and cough appeared within 6 hours, and the elevations of WBC count and CRP level and the enlargement of A-aDO_2_ were present 24 hours after the exposure (Table 2). Chest CT also showed a new ground-glass opacity in the left lower lobe (Fig. 4). Bronchoalveolar lavage fluid (BALF) analysis conducted 24 hours after the exposure revealed elevation of percentages in neutrophils (48%) and lymphocytes (25%) with low CD4/CD8 ratio (0.55), suggesting the acute phase of lung injury. *Acinetobacter spp*. was cultured from the humidifier tank. Taken together, it was proven that her pneumonitis was not provoked by contact with the parakeets but by exposure to the humidifier. Finally, she was diagnosed as having humidifier lung and then no relapse has been experienced after the discard of the humidifier.

**Table 1:** Changes in several parameters before and after the home-returning provocation test.

	Before returning home	6 hours	24 hours
WBC (/μL)	7100	8300	8600
CRP (mg/dL)	0.02	0.02	0.02
A-aDO_2_	6.07	2.70	2.68
KL-6	267	259	263
Body temperature	36.2	36.3	36.3
Change of CT findings		(−)	
Symproms	(−)	(−)	(−)

WBC, White blood cell; CRP, C-reactive protein; A-aDO_2_, alveolar-arterial oxygen difference; CT, Computed tomography.

**Table 2:** Changes in several parameters before and after the humidifier provocation test.

	Before inhalation	6 hours	24 hours
WBC (/μL)	7100	7600	**32,100**
CRP (mg/dL)	0.02	0.02	**2.28**
A-aDO_2_	2.73	7.56	**20.87**
KL-6	255	249	269
Body temperature	36.3	**38.2**	36.3
Change of CT findings			**(+)**
Symptoms	(−)	**Cough, malaise**	(−)

WBC, White blood cell; CRP, C-reactive protein; A-aDO_2_, alveolar-arterial oxygen difference; KL-6, Klebs von den Lungen 6; CT, Computed tomography.

## 3 Discussion

We experienced a case of HP in which two individual provocation tests were helpful to identify the causative exposure. When a home-related antigen is suspected, various inciting agents including fungi, yeasts, or animal proteins are able to be candidates, which makes a diagnosis difficult to specify which one is a culprit exposure. In such cases, the combination of provocation tests can give perspective for diagnosis.

The identification of the causative antigen is crucial in HP for two reasons. First, it apparently prevents a worsening of the patient's prognosis. Missing to identify the causative antigen lead acute HP shifting to recurrent type chronic HP. Pérez et al. reported in a study of 142 patients with HP that the median survival period was significantly longer in the group in which the causative antigen was identified compared to the other group (8 years vs. 2.9 years). They also presented that a failure to identify the causative antigen is an independent predictor of a shorter survival time [4]. The misidentification of inciting antigens results in continuous exposure, which has been associated with poor prognosis due to the development of fibrosis [[5], [6], [7]]. Moreover, advanced stage of fibrotic HP resembles idiopathic pulmonary fibrosis (IPF) radiologically and pathologically and decreases the chance for a correct diagnosis [8]. A diagnostic confidence in HP depends on the likelihood of a causative exposure, which is also needed to distinguish HP from IPF [9,10]. Therefore, the identification of the culprit exposure in the early stage of the disease improves the prognosis of HP. Second, the assessments to find out which antigen is truly culprit may prevent excessive abatement, leading to a decrease in the patient's burden and improve compliance. In our case, the patient had shown a strong attachment for taking care of the parakeet as a hobby. Due to the correct diagnosis of humidifier lung, she had dispensed with giving up keeping the parakeets and with losing her treasure.

However, the causative antigen is not always specifiable only by clinical history and immunological test. In our case, the parakeets and the humidifier were the possible antigens. Although the LST against pigeon serum was positive, the anti-PDE-antibody was negative. This may be due to the fact that the sensitivity of this immunological test is not high [11], and the possibility of bird related HP cannot be denied. On the other hand, a three-month use of a humidifier before disease onset was also suggestive of humidifier lung. An ultrasonic-type humidifier she had used is known to be more possible with contamination of molds and bacteria than thermal-type, which also increase the likelihood of diagnosis as humidifier lung. Thus, both exposure history can be causative for the disease, then we tried a provocation test one by one.

The humidifier provocation test of the present case included one-hour exposure in a hospital room and evaluation at 6 and 24 hours after exposure. Although there are no standardized procedures for the humidifier provocation tests, many of the previous reports show the same protocol as ours [12,13]. However, the timing of the evaluations differ between reports from 6 hours to 24 hours after the exposure. Other than humidifier lung, Inoue et al. reported a significant increase in cytokines including TNF-α, IFN-γ, and IL-6 at 6 hours after inhalation provocation test by PDE antigen in BALF of patients with chronic bird-related HP [14]. The optimal timing of evaluation may be antigen-specific or related to the amount of exposed antigen. Therefore, evaluations at the two time points, 6 and 24 hours post exposure, were set in our case. As a result, only clinical symptoms such as fever and cough were shown in 6 hours and serum inflammatory responses was observed at 24 hours.

In conclusion, we experienced a successfully diagnosed case of HP in which humidifiers or parakeets were simultaneously suspected to be the two causative exposures. A combination of provocation tests was helpful for identification of the inciting antigen.

Learning points:

- For patients with hypersensitivity pneumonitis, it is sometimes difficult to specify the inciting antigen among a wide variety of candidates.
- Exposure provocation tests are useful ways to confirm the culprit antigen, especially when several antigens are simultaneously suspected.
- A thorough medical history-taking is important for making the individual provocation test more relevant.

## Declaration of competing interest

The authors state that they have no conflict of interest.

## References

[1] Costabel U., Miyazaki Y., Pardo A. (2020). Hypersensitivity pneumonitis. Nat. Rev. Dis. Prim..

[2] Miyazaki Y., Tsutsui T., Inase N. (2016). Treatment and monitoring of hypersensitivity pneumonitis. Expet Rev. Clin. Immunol..

[3] Nogueira R., Melo N., Novais E.B.H. (2019). Hypersensitivity pneumonitis: antigen diversity and disease implications. Pulmonology.

[4] Fernández Pérez E.R., Swigris J.J., Forssén A.V. (2013). Identifying an inciting antigen is associated with improved survival in patients with chronic hypersensitivity pneumonitis. Chest.

[5] Ohtani Y., Saiki S., Sumi Y. (2003). Clinical features of recurrent and insidious chronic bird fancier's lung. Ann. Allergy Asthma Immunol..

[6] Yoshizawa Y., Otani Y., Inase N. (2005). [Chronic hypersensitivity pneumonia]. Nihon Naika Gakkai Zasshi.

[7] Kawamoto Y., Oda S., Tanaka M. (2021). Antigen avoidance in people with hypersensitivity pneumonitis: a scoping review. Heart Lung.

[8] Hayakawa H., Shirai M., Sato A. (2002). Clinicopathological features of chronic hypersensitivity pneumonitis. Respirology.

[9] Fernández Pérez E.R., Travis W.D., Lynch D.A. (2021).

[10] Raghu G., Remy-Jardin M., Ryerson C.J. (2020). Diagnosis of hypersensitivity pneumonitis in adults. An official ATS/JRS/ALAT clinical practice guideline. Am. J. Respir. Crit. Care Med..

[11] Suhara K., Miyazaki Y., Okamoto T., Yasui M., Tsuchiya K., Inase N. (2015). Utility of immunological tests for bird-related hypersensitivity pneumonitis. Respir. Investig..

[12] Suda T., Sato A., Ida M., Gemma H., Hayakawa H., Chida K. (1995). Hypersensitivity pneumonitis associated with home ultrasonic humidifiers. Chest.

[13] Imokawa S., Nishimoto K., Suzuki S. (2011). A case of humidifier lung: possible contribution of gram-negative bacteria and fungi. Jpn. J. Sarcoidosis Granulomat. Disord..

[14] Inoue Y., Ishizuka M., Furusawa H. (2019). Acute inflammatory and immunologic responses against antigen in chronic bird-related hypersensitivity pneumonitis. Allergol. Int..

